# Expression of long non-coding RNAs (lncRNAs) has been dysregulated in non-small cell lung cancer tissues

**DOI:** 10.1186/s12885-019-5435-5

**Published:** 2019-03-12

**Authors:** Farbod Esfandi, Mohammad Taheri, Mir Davood Omrani, Mohammad Behgam Shadmehr, Shahram Arsang-Jang, Roshanak Shams, Soudeh Ghafouri-Fard

**Affiliations:** 1 Department of Medical Genetics, Shahid Beheshti University of Medical Sciences, Tehran, Iran; 2 Urogenital Stem Cell Research Center, Shahid Beheshti University of Medical Sciences, Tehran, Iran; 3 Tracheal Diseases Research Center, National Research Institute of Tuberculosis and Lung Diseases (NRITLD), Shahid Beheshti University of Medical Sciences, Tehran, Iran; 4 Clinical Research Development Center (CRDU), Qom University of Medical Sciences, Qom, Iran; 5 Research Center of Gastroenterology and liver Disease, Shahid Beheshti University of Medical Sciences, Tehran, Iran

**Keywords:** Lung cancer, *FAS-AS1*, *GAS5*, *PVT1*, *NEAT1*, *HOTAIRM1*, *TUG1*, *THRIL*

## Abstract

**Background:**

Non-small cell lung cancer (NSCLC) as the most frequent type of lung cancer is associated with extensive mortality. Researchers have studied the suitability of several molecules as biomarkers for early detection of this cancer. Long non-coding RNAs (lncRNAs) as the main regulators of gene expression have also been assessed in this regard.

**Methods:**

In the present study, we compared expression level of *Fas-antisense 1* (*FAS-AS1*), *Growth Arrest Specific 5* (*GAS5*), *PVT1*, *Nuclear Paraspeckle Assembly Transcript 1* (*NEAT1*), *HOXA transcript antisense RNA myeloid-specific 1* (*HOTAIRM1*), *taurine upregulated gene 1* (*TUG1*) and *TNFα and hnRNPL related immunoregulatory LincRNA* (*THRIL*) in 32 NSCLC samples and their corresponding adjacent non-cancerous tissues (ANCTs).

**Results:**

*NEAT1* has been significantly over-expressed in NSCLC tissues obtained from male subjects compared with the corresponding ANCTs (Relative expression (REx) = 3.022, *P* = 0.019) but not in female subjects (*P* = 0.975). *FAS-AS1* was significantly down-regulated in NSCLC tissues obtained from both males and females subjects compared with the corresponding ANCTs (REx = − 4.12 and − 3.14, *P* = 0.015 and 0.033 respectively). *TUG1*, *GAS5*, *THRIL* and *HOTAIRM1* were significantly down-regulated in tumoral tissues obtained from male subjects compared with the corresponding ANCTs.

**Conclusions:**

The observed dysregulation of these lncRNAs in NSCLC tissues compared with the corresponding ANCTs warrants future studies to confirm the results of the current study in larger sample sizes to elaborate their role as cancer biomarkers.

## Background

Lung cancer as the most frequent malignancy and the foremost source of cancer mortality is a heterogeneous disorder. The most common type of lung cancer is non-small-cell lung cancer (NSCLC) which accounts for 85% of the total cases and is further classified into adenocarcinoma, large cell carcinoma and squamous cell carcinoma subtypes [1]. Collectively two thirds of patients with NSCLC are being diagnosed when the tumor is locally advanced or has metastasized [2]. Such delay in the diagnosis of lung cancer in addition to the absence of appropriate therapeutic targets lead to poor patients’ outcome [3]. Consequently, researchers invested substantial efforts in the identification of diagnostic biomarkers and therapeutic targets for this type of human malignancy. Among these putative biomarkers are long non-coding RNAs (lncRNAs) [3]. This proportion of human genome plays fundamental roles in the regulation of tumor suppressor genes and oncogenes expression via epigenetic, transcriptional, and post-transcriptional mechanism [4] and is dysregulated in several human malignancies including NSCLC [5]. A comprehensive study in lung adenocarcinoma has led to identification of 2420 lncRNAs with significant differential expression between tumor and normal tissue samples [6]. Moreover, in silico analysis of NSCLC expression profiles in the Gene Expression Omnibus (GEO) has resulted in recognition of 47 dysregulated lncRNAs in these patients [7]. In addition, dysregulation of lncRNAs in lung cancer tissues has been associated with air pollution [8]. Some well-known risk factors for NSCLC also trigger expression of lncRNAs such as *the smoke and cancer–associated lncRNA–1* (*SCAL1*), *DQ786227*, and *LOC728228* in these tissues [3]. Notably, Wu et al. have detected subtype-dependent lncRNA-associated protein-protein interaction (PPI) modules in human lung cancer and proposed distinct molecular mechanisms for every single subtype. They also demonstrated functional link between antisense lncRNAs and sense genes [9]. Even low ample lncRNAs such as the so-called *Viability Enhancing LUng Cancer Transcript* (*VELUCT*) exert functional roles in the pathogenesis of lung cancer [10]. Other studies have demonstrated aberrant expression of a number lncRNAs including the *Prostate cancer-associated transcript1* (*PCAT1*) [11], *Metastasis-Associated Lung Adenocarcinoma Transcript 1* (*MALAT1*) [12] and *Cancer-Associated Region Long non-coding RNA* (*CARLo-5*) [13] in NSCLC tissues and showed possible links between their expression and malignant features of these cells or patients’ outcomes.

In the present study, in an effort to evaluate the suitability of lncRNAs as biomarkers for NSCLC we compared expression level of seven apoptosis related lncRNAs namely *Fas-antisense 1* (*FAS-AS1*), *Growth Arrest Specific 5* (*GAS5*), *PVT1*, *Nuclear Paraspeckle Assembly Transcript 1* (*NEAT1*), *HOXA transcript antisense RNA myeloid-specific 1* (*HOTAIRM1*), *taurine upregulated gene 1* (*TUG1*) and *TNFα and hnRNPL related immunoregulatory LincRNA* (*THRIL*) in 32 NSCLC samples and their corresponding adjacent non-cancerous tissues (ANCTs) and plotted the receiver operating characteristic (ROC) curve to estimate their appropriateness for classifying disease status. To the best of our knowledge, the current study is the first study to assess relative expression of *HOTAIRM1*, *THRIL* and *FAS-AS1* in lung cancer tissues compared with ANCTs using the quantitative real-time PCR. *NEAT1* is an apoptosis-related lncRNA with remarkable over-expression in plasma samples of NSCLC patients [14]. Contribution of *GAS5* in the pathogenesis of lung cancer has been highlighted through the observed associations between genomic variants within this gene and risk of this malignancy [15]. *TUG1* has been previously shown to exert a tumor suppressor role in NSCLC [16]. Finally, a previous study has suggested a role for *PVT1* in the pathogenesis of NSCLC through inhibition of p15 and p21 expression [17].

In the current investigation, we also assessed the correlation between expression levels of these lncRNAs to find any possible similar regulatory mechanism for these lncRNAs in the context of lung cancer.

## Methods

### Patients’ samples

Cancer samples and the corresponding ANCTs were excised during surgery from 32 patients being admitted at Labbafinejad Hospital with definite diagnosis of NSCLC. None of patients received radiotherapy or chemotherapy before surgery. Tissue samples were transferred to laboratory of Medical Genetics Department in liquid nitrogen. Informed consent forms were obtained from all study participants. The study protocol was approved by the ethical committee of Shahid Beheshti University of Medical Sciences (IR.SBMU.MSP.REC.1395.525). In this study, all methods were performed in accordance with the relevant guidelines and regulations.

### Sampling and RNA extraction

Total RNA was isolated from cancerous tissues and ANCTs using the TRIzol™ Reagent (Invitrogen, Carlsbad, CA, USA) according to the guidelines. The extracted RNA was supposed to DNase I treatment to get rid of DNA contamination. The quantity and quality of the extracted RNA was assessed by Nanodrop equipment (Thermo Scientific) and gel electrophoresis.

### cDNA synthesis and quantitative RT-PCR

cDNA was synthetized from RNA samples using the Applied Biosystems High-Capacity cDNA Reverse Transcription Kit. The relative expression level of each lncRNA was compared between tumoral and non-tumoral tissues using the rotor gene 6000 Corbett Real-Time PCR System. *HPRT1* was used as the reference gene. Primers and probes used for PCR were designed using the Allele ID 7 for × 64 windows software (Premier Biosoft, Palo Alto, USA). The primers and probes sequences and PCR product length are demonstrated in Table 1. Applied Biosystems TaqMan® Universal PCR Master Mix was used for quantification of lncRNAs expression. PCR program included a denaturation step at 95 °C for 10 min, followed by 40 cycles of 95 °C for 10 s and 60 °C for 60 s and a final extension step in 72 °C for 5 min.

**Table 1:** The primers and probes sequences and PCR product length

Gene name	Primer and probe sequence	Primer and probe length	Product length
*HPRT1*	F: AGCCTAAGATGAGAGTTC	18	88
	R: CACAGAACTAGAACATTGATA	21	
	FAM -CATCTGGAGTCCTATTGACATCGC- TAMRA	24	
*NEAT1*	F: CCAGTGTGAGTCCTAGCATTGC	20	78
	R: CCTGGAAACAGAACATTGGAGAAC	22	
	FAM- ACCCTGGAGGAGAGAGCCCGCC - TAMRA	23	
*TUG1*	F: ACCGGAGGAGCCATCTTGTC	24	149
	R: GAAAGAGCCGCCAACCGATC	24	
	FAM - ACCGCACGCCCGTTCCTTCGC -TAMRA	24	
*FAS-AS1*	F: GAAAAGGTGCCGTTCTTCCG	20	81
	R: CTGGCAGTTCTCAGACGTAGG	20	
	FAM - CGGCTTAACCACTGCTTCGGTGCT -TAMRA	23	
*GAS5*	F: CTGCTTGAAAGGGTCTTGCC	23	91
	R: GGAGGCTGAGGATCACTTGAG	23	
	FAM- ACCCAAGCTAGAGTGCAGTGGCCT- TAMRA	24	
*PVT1*	F: CCCATTACGATTTCATCTC	20	131
	R: GTTCGTACTCATCTTATTCAA	21	
	FAM- AGCAAGCACCTGTTACCTGTC - TAMRA	20	
*HOTAIRM1*	F: GAAGAGCAAAAGCTGCGTTCTG	22	135
	R: CTCTCGCCAGTTCATCTTTCATTG	24	
	FAM-CCCGACTCCGCTGCCCGCCC-TAMRA	20	
*THRIL*	F: GAGTGCAGTGGCGTGATCTC	20	121
	R: AAAATTAGTCAGGCATGGTGGTG	20	
	FAM- CTCACCGCAACCTCCACCTCCCAG- TAMRA	23	

### Statistical analysis

Relative expression of lncRNAs in tumoral tissues compared with ANCTs was estimated based on calculation of Ln [Efficiency^∆CT] values. The association between lncRNAs transcript levels and clinicipathologic data of patients was evaluated using Chi-square test. Spearman rank order correlation test was used to estimate the correlation between relative expression levels of lncRNAs and patients’ age. Statistical analyses were performed in R 3.5.1. The effects of possible confounding variables such as age and sex with were assessed using the Quantile regression model. Differences between tumoral and ANCTs were analyzed using Bayesian modeling in RStan using brms and BEST package with Iteration = 5000 and Warmup = 2000. Convergence was assessed using Rhat parameter. *P* values less than 0.05 were considered significant.

The receiver operating characteristic (ROC) curve was plotted to evaluate the suitability of gene expression levels for classifying disease status. In order to estimate gene expression probability cut-off the Youden index (j) was used to maximize the difference between sensitivity (true-positive rate) and 1 – specificity (false-positive rate). The accuracy of each marker for diagnosis of lung cancer was scored based on the area under curve (AUC) values using the following system: 0.90–1 = excellent (A), 0.80–0.90 = good (B), 0.70–0.80 = fair (C), 0.60–0.70 = poor (D) and 0.50–0.60 = fail (F).

### In silico analyses

We used LncRNAtor online tool [18] to assess target genes of lncRNAs in lung cancer tissues. The retrieved target genes were scored based on r and *P* values and those with r > 0.2 and *P* < 0.05 were subjected to further Gene Ontology (GO) and Kyoto Encyclopedia of Genes and Genomes (KEGG) pathway enrichment analysis by DAVID 6.8 tool (https://david.ncifcrf.gov/summary.jsp). Finally, we assessed lncRNAs targets at protein level by using starBase v2.0 [19]. The interaction network between theses lncRNAs and their targets was depicted using Gene MANIA tool [20].

## Results

### General clinical and demographic data of patients

The mean age *of* study participants was 57.96 ± 7.73 years, ranging from 37 to 80 years. Other features are shown in Table 2.

**Table 2:** General data of NSCLC patients

Gender *N* (%)		Smoking *N* (%)		Subtype *N* (%)		Stage *N* (%)		
Male	Female	Yes	No	Adenocarcinoma	Squamous cell carcinoma	1	2	3
24 (75)	8 (25)	6 (18.75)	26 (81.25)	18 (56.25)	14 (43.75)	7 (21.88)	11 (34.38)	14 (43.75)

### Relative expression of lncRNAs in tumoral tissues vs. ANCTs

**Fig. 1:**
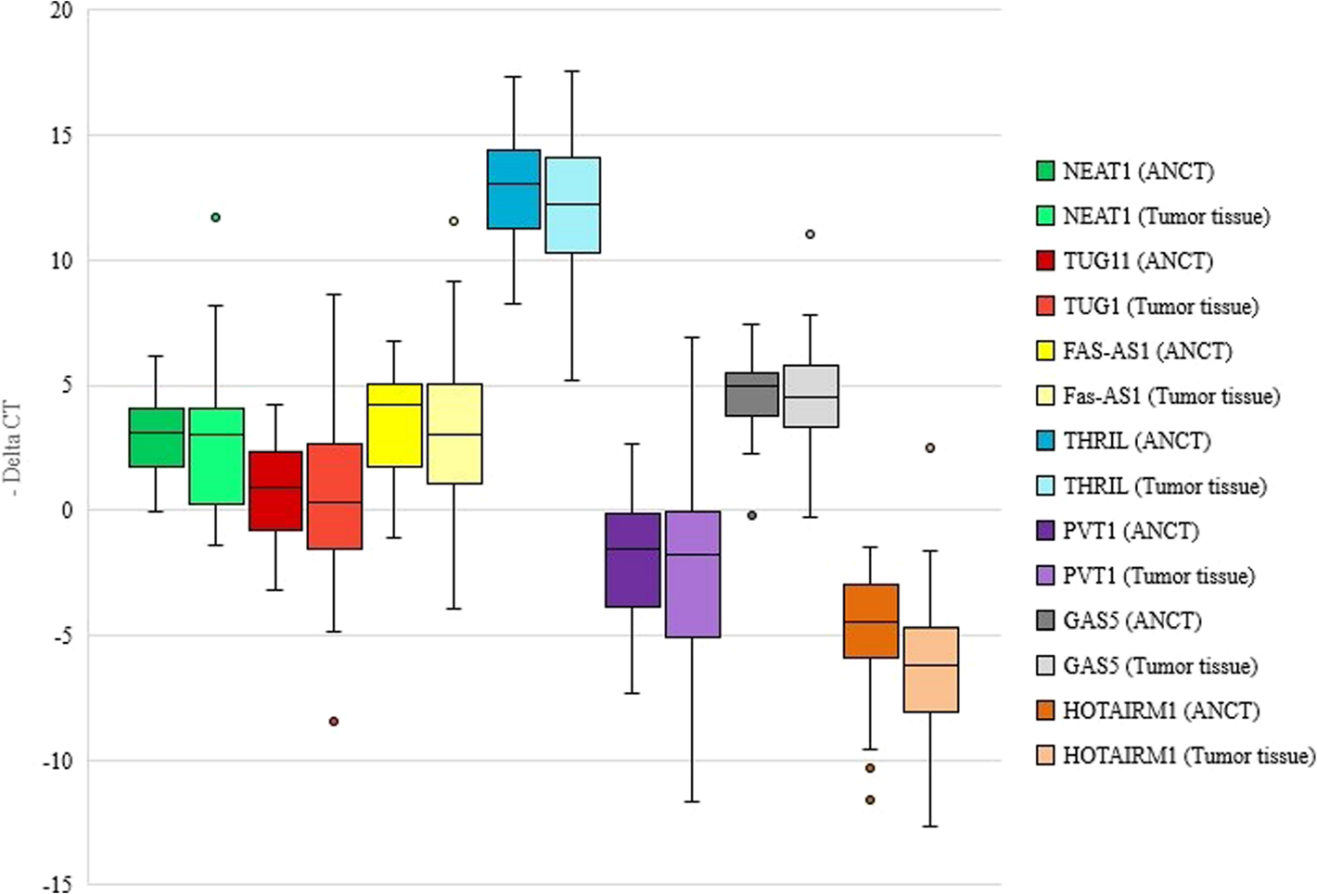
Relative expression of lncRNAs in NSCLC samples and ANCTs

Among the lncRNAs, *NEAT1* was the only up-regulated lncRNA in tumoral tissues while *GAS5* had the highest down-regulation in tumoral tissues compared with ANCTs. *NEAT1* has been significantly over-expressed in NSCLC tissues obtained from male subjects compared with the corresponding ANCTs (Relative expression (REx) = 3.022, *P* = 0.019) but not in female subjects (*P* = 0.975). *FAS-AS1* was significantly down-regulated in NSCLC tissues obtained from both males and females subjects compared with the corresponding ANCTs (REx = − 4.12 and − 3.14, *P* = 0.015 and 0.033 respectively). *TUG1*, *GAS5*, *THRIL* and *HOTAIRM1* were significantly down-regulated in tumoral tissues obtained from male subjects compared with the corresponding ANCTs (Table 3). Figure 1 shows relative expression of lncRNAs in tumor tissues and ANCTs.

**Table 3:** Relative expression of lncRNAs in tumoral tissues compared with ANCTs (REx: Relative expression based of Ln [Efficiency^∆CT] values, SE: Standard Error, 95% Crl: 95% Credible Interval, *P-values and related confidence Intervals estimated using Bonferroni correction)

	Total samples					Tissue samples from male patients					Tissue samples from female patients				
	REx	SE	Effect Size	*P*-value	95% CrI	REx	SE	Effect Size	*P*-value*	95% CrI*	REx	SE	Effect Size	*P*-value*	95% CrI*
*NEAT1*	2.218	2.216	0.398	0.026	[0.14, 4.32]	3.027	1.33	0.502	0.038	[0.15, 5.92]	0.063	1.84	0.0145	> 0.999	[−3.93, 4.06]
*TUG1*	−2.798	−2.8	−0.744	< 0.0001	[−4.19, −1.38]	−2.76	0.881	−0.694	0.004	[−4.68, − 0.85]	− 2.971	1.6	− 0.868	0.126	[−6.45, 0.51]
*FAS-AS1*	−3.95	1.1	−0.759	0.002	[−6.14, − 1.76]	− 4.12	1.47	− 0.68	0.03	[−7.31, − 0.94]	− 3.147	1.58	− 0.855	0.066	[− 6.58, 0.29]
*GAS5*	−5.307	1.01	− 0.997	< 0.0001	[−7.34, − 3.36]	−5.6	1.19	−1.04	< 0.0001	[−8.19, − 3.02]	− 4.48	2.64	−0.751	0.136	[− 10.21, 1.25]
*PVT1*	− 2.123	− 2.11	− 0.404	0.034	[−4.15, − 0.19]	− 2.104	1.15	− 0.403	0.128	[− 4.6, 0.4]	−2.171	2.72	− 0.354	0.708	[− 8.08, 3.74]
*THRIL*	−2.542	0.82	− 0.583	0.002	[−4.18, − 0.94]	− 2.93	.995	− 0.65	0.006	[−5.09, − 0.78]	−1.405	1.93	− 0.324	0.816	[− 5.6, 2.79]
*HOTAIRM1*	− 2.347	0.71	−0.622	0.001	[− 3.74, − 0.93]	−2.381	.86	−0.608	0.012	[−4.25, − 0.52]	− 2.285	1.72	− 0.599	0.3	[−6.02, 1.45]

### Association study of lncRNAs expression levels and clinicopathological data of patients

No significant association was found between expression levels of mentioned lncRNAs and patients’ clinicopathologic data when dividing patients into down−/up-regulation categories based on relative expression of each lncRNA in tumoral tissue compared with the paired ANCT (Table 4). However, a significant association was found between relative expression of *TUG1* and cancer subtype (Table 5).

**Table 4:** Association study of lncRNAs expression and clinicopathological data of patients

	*FAS-AS1* up-regulation	*FAS-AS1* down-regulation	P value	*HOTAIRM1* up-regulation	*HOTAIRM1* Down-regulation	P value	*NEAT1* up-regulation	*NEAT1* down-regulation	P value	*PVT1* up-regulation	*PVT1* down-regulation	P value	*THRIL* up-regulation	*THRIL* down-regulation	P value	*TUG1* up-regulation	*TUG1* down-regulation	P value	*GAS5* up-regulation	*GAS5* down-regulation	P value
Age			1			0.48			0.48			0.723			0.476			0.723			0.723
< 60 years	8(50%)	8(50%)		9 (56.3%)	7(43.8%)		9(56.2%)	7(43.8)		9(56.3%)	7(43.8%)		10(62.5%)	6(37.5%)		9(56.2%)	7(43.8)		7(43.8%)	9(56.3%)	
≥60 years	8(50%)	8(50%)		7(43.8%)	9(56.3%)		7(43.8)	9(56.2%)		8(50%)	8(50%)		8(50%)	8(50%)		8(50%)	8(50%)		8(50%)	8(50%)	
Smoking			1			1			1			1			0.426			0.272			0.678
Yes	13(52%)	12(48%)		12(48%)	13(52%)		12(48%)	13(52%)		13(52%)	12(48%)		13(52%)	12(48%)		12(48%)	13(52%)		11(44%)	14(56%)	
No	3(42.9%)	4(57.1%)		4(57.1%)	3(42.9%)		4(57.1%)	3(42.9%)		4(57.1%)	3(42.9%)		5(71.4%)	2(28.6%)		5(71.4%)	2(28.6%)		4(57.1%)	3(42.9%)	
Stage			0.148			0.148			0.538			0.73			1			0.165			0.39
1	6(85.7%)	1 (14.3%)		6(85.7%)	1 (14.3%)		5(71.4%)	2(28.6%)		3(42.9%)	4(57.1%)		4(57.1%)	3(42.9%)		6(85.7%)	1 (14.3%)		5(71.4%)	2(28.6%)	
2	4(36.4%)	7(63.6%)		4(36.4%)	7(63.6%)		5(45.5%)	6(54.5%)		7(63.6%)	4(36.4%)		6(54.5%)	5(45.5%)		5(45.5%)	6(54.5%)		4(36.4%)	7(63.6%)	
3	6(42.9%)	8(57.1%)		6(42.9%)	8(57.1%)		6(42.9%)	8(57.1%)		7(50%)	7(50%)		8(57.1%)	6(42.9%)		6(42.9%)	8(57.1%)		6(42.9%)	8(57.1%)	
Subtype			0.476			1			1			0.688			0.53			0.688			0.305
Adenocarcinoma	8(44.8%)	10(55.6%)		9 (50%)	9 (50%)		9 (50%)	9 (50%)		9 (50%)	9 (50%)		11(61.1%)	7(38.9%)		9 (50%)	9 (50%)		7(38.9%)	11(61.1%)	
Squamous cell carcinoma	8(57.1%)	6 (42.9%)		7 (50%)	7 (50%)		7 (50%)	7 (50%)		8 (57.1%)	9 (42.9%)		7 (50%)	7 (50%)		8 (57.1%)	6 (42.9%)		8(57.1%)	6 (42.9%)

**Table 5:** Association between the relative expression of lncRNAs and independent variables

lncRNAs	Parameters	Beta	SE	t	P-value	95% CI for Beta
*NEAT1*	Age	0.04	0.13	0.28	0.78	[−0.24, 0.31]
	Gender (Female/Male)	−1.62	2.33	−0.70	0.49	[−6.42, 3.17]
	Smoking (Yes/No)	− 0.84	2.63	−0.32	0.75	[−6.26, 4.57]
	Subtype (SCC/Adeno)	−2.64	1.99	−1.32	0.20	[−6.74, 1.46]
	Stage					
	2	0.71	2.62	0.27	0.79	[−4.69, 6.1]
	3	2.70	2.41	1.12	0.27	[−2.26, 7.66]
*TUG1*	Age	0.19	0.11	1.64	0.11	[−0.05, 0.42]
	Gender (Female/Male)	−3.78	2.00	−1.89	0.07	[−7.89, 0.33]
	Smoking (Yes/No)	1.74	2.26	0.77	0.45	[−2.91, 6.39]
	Subtype (SCC/Adeno)	−3.84	1.71	−2.25	0.03	[−7.36, −0.32]
	Stage					
	2	0.05	2.25	0.02	0.98	[−4.57, 4.68]
	3	0.84	2.06	0.41	0.69	[− 3.41, 5.09]
*FAS-AS1*	Age	0.06	0.15	0.37	0.71	[−0.26, 0.38]
	Gender (Female/Male)	2.23	2.71	0.82	0.42	[−3.34, 7.81]
	Smoking (Yes/No)	−0.50	3.06	−0.16	0.87	[−6.81, 5.81]
	Subtype (SCC/Adeno)	−0.71	2.32	−0.31	0.76	[−5.49, 4.06]
	Stage					
	2	−0.61	3.05	−0.20	0.84	[−6.89, 5.67]
	3	0.88	2.80	0.32	0.76	[−4.88, 6.65]
*GAS5*	Age	0.14	0.13	1.06	0.30	[−0.14, 0.42]
	Gender (Female/Male)	−0.84	2.36	−0.36	0.72	[−5.7, 4.02]
	Smoking (Yes/No)	−2.55	2.67	−0.96	0.35	[−8.05, 2.95]
	Subtype (SCC/Adeno)	−3.73	2.02	−1.84	0.08	[−7.89, 0.44]
	Stage					
	2	3.32	2.66	1.25	0.22	[−2.16, 8.79]
	3	3.52	2.44	1.44	0.16	[−1.51, 8.55]
*PVT1*	Age	0.17	0.13	1.27	0.22	[−0.1, 0.44]
	Gender (Female/Male)	−0.66	2.31	−0.29	0.78	[−5.42, 4.1]
	Smoking (Yes/No)	−1.36	2.62	−0.52	0.61	[−6.74, 4.03]
	Subtype (SCC/Adeno)	−1.74	1.98	−0.88	0.39	[−5.81, 2.34]
	Stage					
	2	0.69	2.60	0.27	0.79	[−4.67, 6.05]
	3	1.14	2.39	0.48	0.64	[−3.78, 6.07]
*THRIL*	Age	0.01	0.16	0.07	0.95	[−0.33, 0.35]
	Gender (Female/Male)	0.52	2.87	0.18	0.86	[−5.39, 6.44]
	Smoking (Yes/No)	0.00	3.25	0.00	>.999	[−6.69, 6.69]
	Subtype (SCC/Adeno)	−2.77	2.46	−1.13	0.27	[−7.84, 2.29]
	Stage					
	2	−3.20	3.23	−0.99	0.33	[−9.86, 3.46]
	3	0.19	2.97	0.07	0.95	[−5.93, 6.31]
*HOTAIRM1*	Age	0.46	0.24	1.94	0.06	[−0.03, 0.96]
	Gender (Female/Male)	−1.92	4.19	−0.46	0.65	[−10.55, 6.72]
	Smoking (Yes/No)	3.57	4.74	0.75	0.46	[−6.19, 13.33]
	Subtype (SCC/Adeno)	− 6.50	3.59	−1.81	0.08	[−13.89, 0.89]
	Stage					
	2	0.64	4.72	0.14	0.89	[−9.08, 10.36]
	3	1.24	4.34	0.29	0.78	[−7.68, 10.17]

### Correlation analysis between expression levels of lncRNAs in tumoral tissues and ANCTs

Spearman Correlation analysis revealed significant correlations between relative expression levels of lncRNAs especially within tumor tissues and in male subgroup (Table 6).

**Table 6:** Correlations between relative expression levels of lncRNAs in tumoral tissues and ANCTs based on patients’ sex (When dividing patients based on their sex, both tumor tissues and ANCTs were assessed)

		*FAS-AS1*	*GAS5*	*PVT1*	*NEAT1*	*HOTAIRM1*	*TUG1*
*THRIL*	Male	.639^a^	.770^a^	.524^a^	.585^a^	.455^a^	.549^a^
	Female	.603^b^	.653^a^	.244	.403	.594^b^	.412
	Tumor	.601^a^	.784^a^	.326	.576^a^	.387^b^	.498^a^
	ANCT	.318	.447^b^	.32	.495^a^	.353^b^	.335
*TUG1*	Male	.574^a^	.568^a^	.342^b^	.471^a^	.459^a^	
	Female	.638^a^	.812^a^	.612^b^	.394	.506^b^	
	Tumor	.606^a^	.75^a^	.464^a^	.687^a^	.464^a^	
	ANCT	.282	.170	0.53	.181	.345	
*HOTAIRM1*	Male	.491^a^	.408^a^	.395^a^	.470^a^		
	Female	.509^b^	.565^b^	.185	.209		
	Tumor	.432^b^	.533^a^	.266	.446^b^		
	ANCT	.221	.052	.388^b^	.333		
*NEAT1*	Male	.623^a^	.731^a^	.519^a^			
	Female	.424	.418	.532^b^			
	Tumor	.749^a^	.785^a^	.746^a^			
	ANCT	.282	.529^a^	.125			
*PVT1*	Male	.468^a^	.345^b^				
	Female	.456	.703^a^				
	Tumor	.622^a^	.699^a^				
	ANCT	.099	.028				
*GAS5*	Male	.770^a^					
	Female	.653^a^					
	Tumor	.784^a^					
	ANCT	.447^b^					

^a^Correlation is significant at the 0.01 level

^b^Correlation is significant at the 0.05 level

### ROC curve analysis

**Fig. 2:**
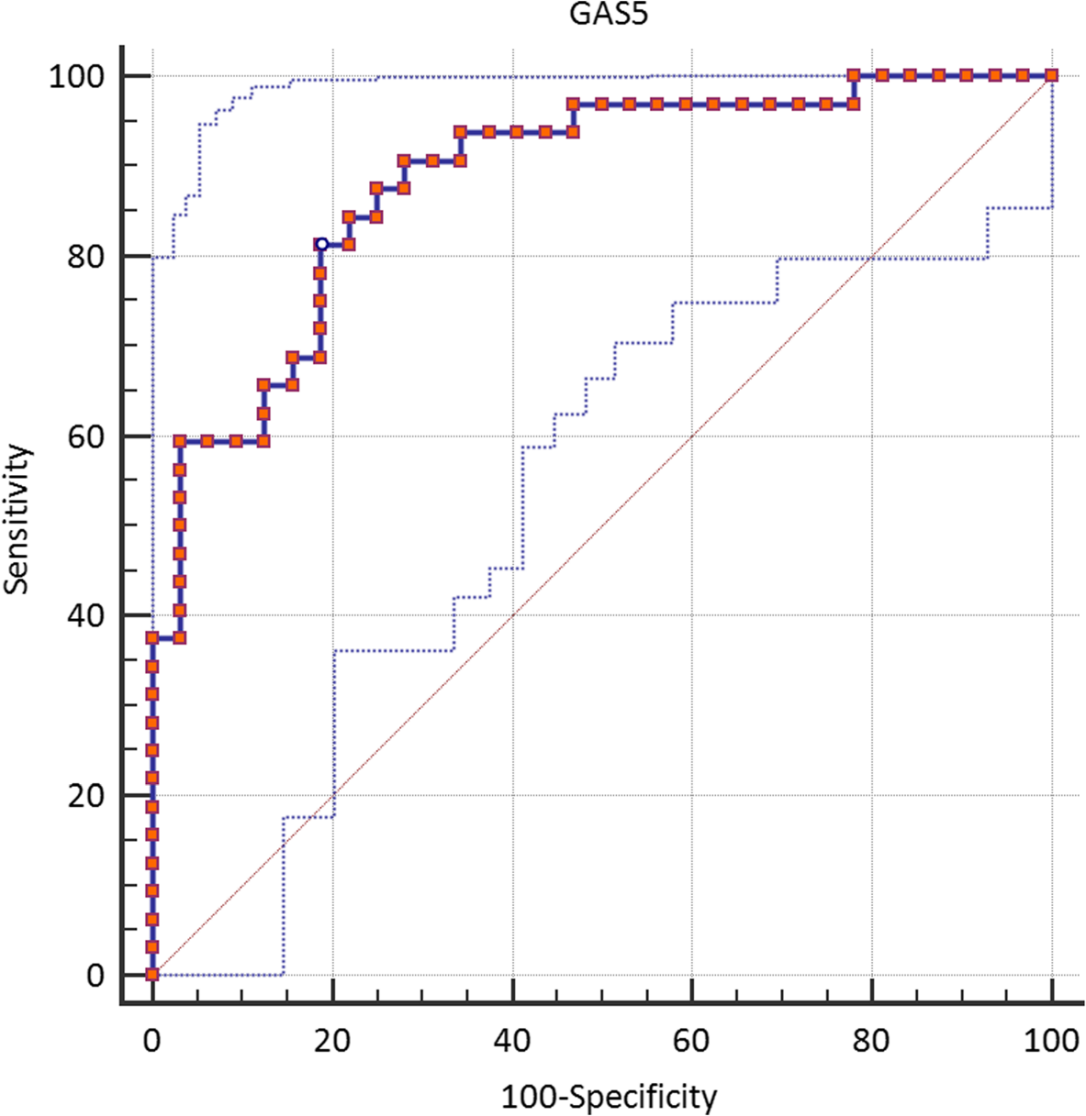
ROC curve analysis for *GAS5*

Based on ROC curve analysis results, the accuracy of *GAS5* expression levels for lung cancer diagnosis is good (Fig. 2). Besides, *TUG1*, *FAS-AS1* and *THRIL* expression levels were fair diagnostic markers for lung cancer. Table 7 shows the details of ROC curve analysis.

**Table 7:** The results of ROC curve analysis (^a^Youden index, ^b^Significance level P (Area = 0.5), Estimate criterion: optimal cut-off point for gene expression (ln(E^CT^_reffrence_/E^Ct^_target_))

	Estimate criterion	AUC	J^a^	Sensitivity	Specificity	*P*-value^b^
*NEAT1*	> 0.13	0.676	0.312	75	56.25	0.008
*TUG1*	≤0.191	0.715	0.437	53.13	90.62	0.001
*FAS-AS1*	≤ − 2.82	0.764	0.5	59.38	90.62	< 0.0001
*GAS5*	≤ − 1.991	0.884	0.625	81.25	81.25	< 0.0001
*PVT1*	≤1.69	0.649	0.281	65.62	62.5	0.032
*THRIL*	<−7.22	0.705	0.375	53.13	84.37	0.002
*HOTAIRM1*	≤ − 2.203	0.624	0.2813	31.25	96.87	0.081

**Fig. 3:**
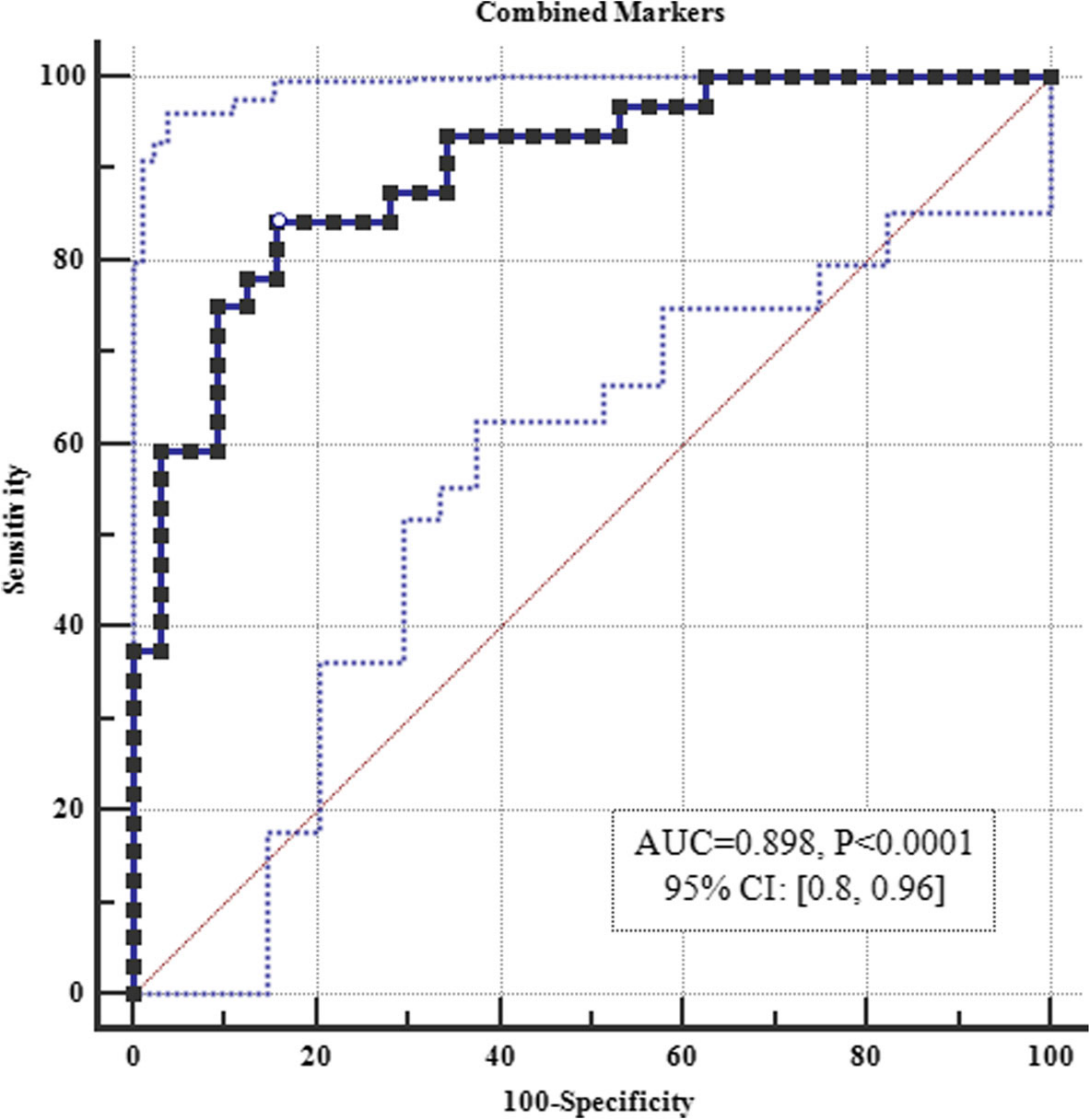
ROC curve analysis for combination of differentially expressed lncRNAs

We also combined all differentially expressed lncRNAs in ROC curve analysis. This method raised the diagnostic power to 0.898 based on the obtained AUC value (Fig. 3).

### KEGG pathway enrichment analysis

KEGG pathway enrichment analysis showed the targeted genes participate in a number of cancer-related pathways such as chemokine signaling, HIF-1, JAK-STAT and NOTH and thyroid hormone signaling pathways as well as some virus-associated pathways. Table 8 shows the results of KEGG pathway enrichment analysis.

**Table 8:** The results of KEGG pathway enrichment analysis of lncRNAs target genes

Term ID	Description	Genes	Count	%	*P*-value	False Discovery Rate
hsa05200	Pathways in cancer	AKT1, BRAF, BCR, CREBBP, CRKL, KIT, TRAF3, ADCY4,ARNT, AXIN1, CTNNA1, COL4A3, CYCS, FZD6, GSK3B, LAMA4, LAMC1, PIK3R2, PTGER2, RBX1, STAT3, STAT5A, TCEB1	23	12.4	1.10E-05	2.50E-03
hsa05169	Epstein-Barr virus infection	AKT1,CREBBP, POLR2H, POLR3C, POLR3K, TRAF3, XPO1, GSK3B, PIK3R2, PSMC6, PSMD11, PSMD14, STAT3, YWHAG	14	7.6	1.10E-04	8.30E-03
hsa04110	Cell cycle	BUB1, BUB3, CREBBP, ANAPC11, ANAPC2, CCNB1, GSK3B, ORC2, ORC3, RBX1, SMC3, YWHAG	12	6.5	3.80E-05	4.10E-03
hsa05166	HTLV-I infection	AKT1, BUB3, CREBBP, POLE, ADCY4, ANAPC11, ANAPC2, XPO1, FZD6, GSK3B, PIK3R2, STAT5A	12	6.5	1.50E-02	1.50E-01
hsa03010	Ribosome	RPL12, RPL30, RPL35, RPL37A, RPL38, RPL4, RPL8, RPS5, RPS7, RPS8, RPLP1	11	5.9	4.10E-04	1.80E-02
hsa05164	Influenza A	AKT1, CREBBP, CYCS, XPO1, GSK3B, HNRNPUL1, PIK3R2, PABPN1, RAE1, SOCS3, TLR4	11	5.9	2.70E-03	7.10E-02
hsa03013	RNA transport	RANBP2, RBM8A, UPF3A, EIF3J, EIF5B, XPO1, GEMIN4, GEMIN6, NUP205, RAE1	10	5.4	8.00E-03	1.10E-01
hsa05205	Proteoglycans in cancer	AKT1, BRAF, IQGAP1, TIAM1, FZD6, PIK3R2, PPP1CC, PTPN6, STAT3, TLR4	10	5.4	2.00E-02	1.70E-01
hsa04932	Non-alcoholic fatty liver disease	AKT1, NDUFB9, COX5A, COX7C, CYCS, GSK3B, PIK3R2, PRKAG1, SOCS3	9	4.9	1.10E-02	1.30E-01
hsa04062	Chemokine signaling pathway	AKT1, BRAF, CRKL, TIAM1, ADCY4, GSK3B, PIK3R2, PRKCD, STAT3	9	4.9	3.50E-02	2.30E-01
hsa04510	Focal adhesion	AKT1, BRAF, CRKL, COL4A3, GSK3B, LAMA4, LAMC1, PIK3R2, PPP1CC	9	4.9	5.70E-02	3.10E-01
hsa04066	HIF-1 signaling pathway	AKT1, CREBBP, ARNT, PIK3R2, RBX1, STAT3, TLR4, TCEB1	8	4.3	3.60E-03	8.30E-02
hsa04919	Thyroid hormone signaling pathway	AKT1, CREBBP, GSK3B, MED13, NOTCH2, NOTCH4, NCOR1, PIK3R2	8	4.3	8.20E-03	1.00E-01
hsa05222	Small cell lung cancer	AKT1, TRAF3, COL4A3, CYCS, LAMA4, LAMC1, PIK3R2	7	3.8	7.30E-03	1.10E-01
hsa04660	T cell receptor signaling pathway	AKT1, CD4, GSK3B, LCP2, PIK3R2, PTPN6, PTPRC	7	3.8	1.80E-02	1.60E-01
hsa04068	FoxO signaling pathway	AKT1, BRAF, CREBBP, CCNB1, PIK3R2, PRKAG1, STAT3	7	3.8	5.40E-02	3.10E-01
hsa04630	Jak-STAT signaling pathway	AKT1, CREBBP, PIK3R2, PTPN6, STAT3, STAT5A, SOCS3	7	3.8	7.40E-02	3.40E-01
hsa03015	mRNA surveillance pathway	RBM8A, SMG1, SMG5, UPF3A, PABPN1, PPP1CC	6	3.2	3.70E-02	2.40E-01
hsa04750	Inflammatory mediator regulation of TRP channels	ADCY4, PIK3R2, PLA2G4F, PTGER2, PRKCD, PPP1CC	6	3.2	4.80E-02	2.90E-01
hsa04330	Notch signaling pathway	CREBBP, MAML2, NOTCH2, NOTCH4	4	2.2	7.30E-02	3.50E-01

### GO analysis of differentially expressed target genes of lncRNAs in lung cancer

The lncRNAs target genes are involved in cancer-related cellular processes such as cell cycle control, cell division, translation and signal transduction (Table 9).

**Table 9:** GO analysis of differentially expressed target genes of lncRNAs in lung cancer

Category	Term	Count	%	*P*-value	False Discovery Rate
GOTERM_BP_DIRECT	GO:0007062~sister chromatid cohesion	29	15.7	4.40E-05	5.60E-03
GOTERM_BP_DIRECT	GO:0000184~nuclear-transcribed mRNA catabolic process, nonsense-mediated decay	15	8.1	4.80E-12	6.70E-09
GOTERM_BP_DIRECT	GO:0019083~viral transcription	15	8.1	3.50E-11	2.50E-08
GOTERM_BP_DIRECT	GO:0006614~SRP-dependent cotranslational protein targeting to membrane	15	8.1	3.10E-03	1.20E-01
GOTERM_BP_DIRECT	GO:0006413~translational initiation	14	7.6	2.20E-10	1.00E-07
GOTERM_BP_DIRECT	GO:0051056~regulation of small GTPase mediated signal transduction	14	7.6	2.10E-05	3.30E-03
GOTERM_BP_DIRECT	GO:0006364~rRNA processing	14	7.6	1.10E-04	1.10E-02
GOTERM_BP_DIRECT	GO:0006412~translation	13	7	2.80E-08	7.80E-06
GOTERM_BP_DIRECT	GO:0016032~viral process	13	7	3.40E-06	6.90E-04
GOTERM_BP_DIRECT	GO:0006367~transcription initiation from RNA polymerase II promoter	13	7	1.90E-05	3.30E-03
GOTERM_BP_DIRECT	GO:0007165~signal transduction	12	6.5	5.00E-09	1.80E-06
GOTERM_BP_DIRECT	GO:0000132~establishment of mitotic spindle orientation	11	5.9	1.80E-06	4.20E-04
GOTERM_BP_DIRECT	GO:0051301~cell division	11	5.9	5.00E-02	5.90E-01
GOTERM_BP_DIRECT	GO:0051436~negative regulation of ubiquitin-protein ligase activity involved in mitotic cell cycle	11	5.9	5.10E-02	5.90E-01
GOTERM_BP_DIRECT	GO:0043488~regulation of mRNA stability	11	5.9	8.50E-02	7.10E-01
GOTERM_BP_DIRECT	GO:0045860~positive regulation of protein kinase activity	10	5.4	3.90E-05	5.40E-03
GOTERM_BP_DIRECT	GO:0051437~positive regulation of ubiquitin-protein ligase activity involved in regulation of mitotic cell cycle transition	10	5.4	1.50E-03	7.30E-02
GOTERM_BP_DIRECT	GO:0032869~cellular response to insulin stimulus	10	5.4	3.00E-02	4.70E-01
GOTERM_BP_DIRECT	GO:0031145~anaphase-promoting complex-dependent catabolic process	10	5.4	5.70E-02	6.00E-01
GOTERM_BP_DIRECT	GO:0016925~protein sumoylation	9	4.9	1.60E-03	7.60E-02
GOTERM_BP_DIRECT	GO:0007052~mitotic spindle organization	9	4.9	2.80E-03	1.10E-01
GOTERM_BP_DIRECT	GO:1990090~cellular response to nerve growth factor stimulus	8	4.3	1.20E-04	1.10E-02
GOTERM_BP_DIRECT	GO:0071407~cellular response to organic cyclic compound	8	4.3	2.70E-04	1.90E-02
GOTERM_BP_DIRECT	GO:0006406~mRNA export from nucleus	8	4.3	2.30E-02	4.20E-01
GOTERM_BP_DIRECT	GO:0000082~G1/S transition of mitotic cell cycle	8	4.3	9.90E-02	7.50E-01
GOTERM_BP_DIRECT	GO:1900034~regulation of cellular response to heat	7	3.8	1.10E-04	1.10E-02
GOTERM_BP_DIRECT	GO:0007067~mitotic nuclear division	7	3.8	1.60E-04	1.40E-02
GOTERM_BP_DIRECT	GO:0043161~proteasome-mediated ubiquitin-dependent protein catabolic process	7	3.8	1.80E-04	1.40E-02
GOTERM_BP_DIRECT	GO:0006297~nucleotide-excision repair, DNA gap filling	7	3.8	2.00E-04	1.50E-02
GOTERM_BP_DIRECT	GO:1901796~regulation of signal transduction by p53 class mediator	7	3.8	7.20E-04	4.20E-02
GOTERM_BP_DIRECT	GO:0006368~transcription elongation from RNA polymerase II promoter	7	3.8	8.00E-04	4.40E-02
GOTERM_BP_DIRECT	GO:0000070~mitotic sister chromatid segregation	7	3.8	2.20E-03	9.80E-02
GOTERM_BP_DIRECT	GO:0000398~mRNA splicing, via spliceosome	7	3.8	3.60E-03	1.30E-01
GOTERM_BP_DIRECT	GO:0043547~positive regulation of GTPase activity	7	3.8	5.20E-03	1.70E-01
GOTERM_BP_DIRECT	GO:0061418~regulation of transcription from RNA polymerase II promoter in response to hypoxia	7	3.8	6.50E-03	1.90E-01
GOTERM_BP_DIRECT	GO:0006661~phosphatidylinositol biosynthetic process	7	3.8	8.30E-03	2.30E-01

**Fig. 4:**
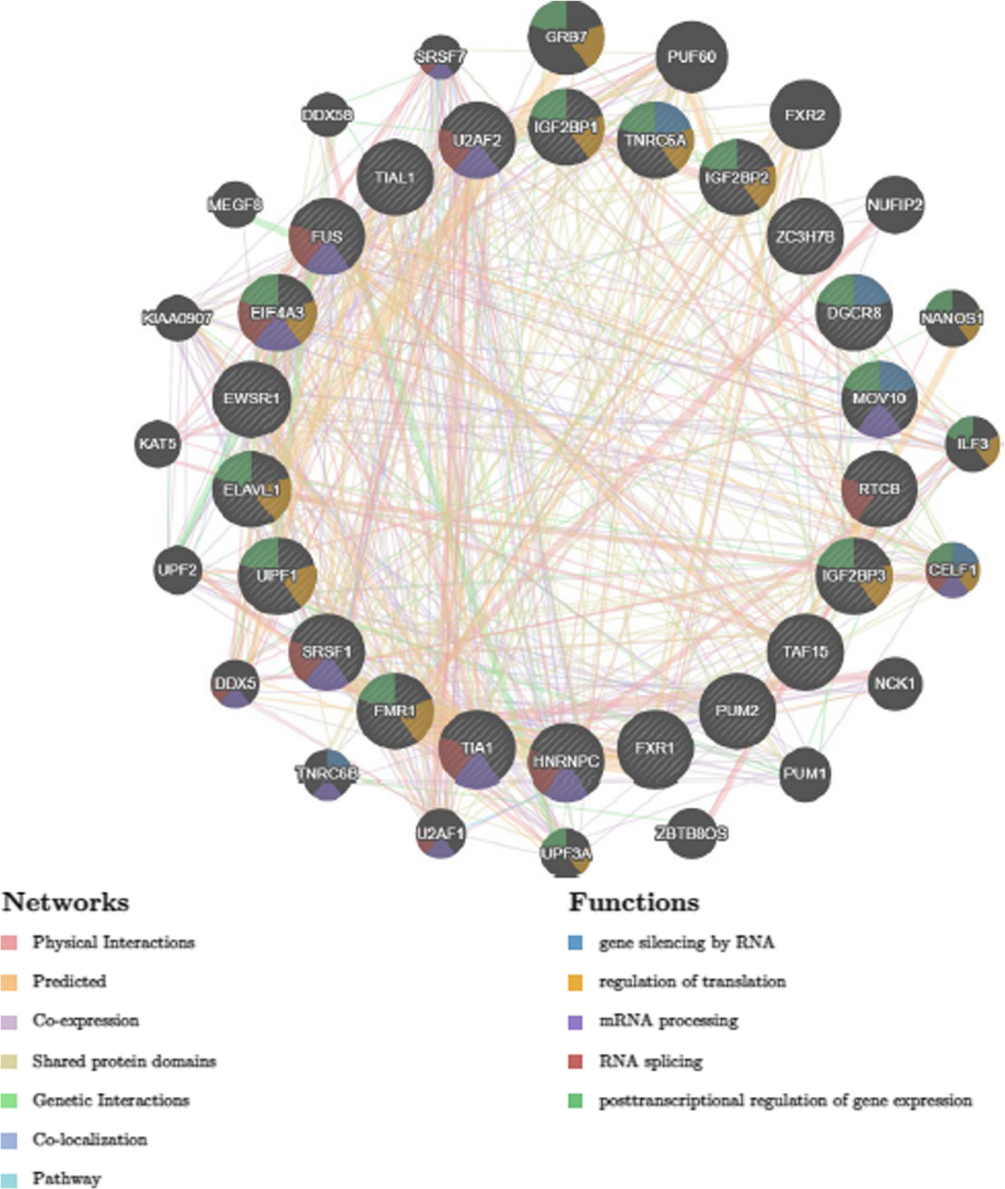
Analysis of interaction network between these lncRNAs and their targets showed that the enriched pathways were related to gene silencing by RNA, regulation of translation, mRNA processing, RNA splicing and posttranscriptional regulation of gene expression

Finally, we provided a list of differentially expressed target proteins of lncRNAs in lung cancer using starBase tool (Table 10) and depicted the network between these lncRNAs and their targets (Fig. 4). The enriched pathways were related to gene silencing by RNA, regulation of translation, mRNA processing, RNA splicing and posttranscriptional regulation of gene expression.

**Table 10:** Differentially expressed target proteins of lncRNAs in lung cancer

	r values		*P* values	
	adenocarcinoma	Squamous cell carcinoma	adenocarcinoma	Squamous cell carcinoma
GAS5 protein targets				
IGF2BP2	−0.32657	NS	4.38064e-15	NS
TNRC6	−0.24924	−0.09044	3.3202e-09	0.036
eIF4AIII	0.23814	0.24445	1.66706e-08	1.08209e-08
FXR1	NS	0.21644	NS	4.53143e-07
ZC3H7B	−0.39888	− 0.3438	2.41224e-22	3.11115e-16
TIA1	0.30713	0.36671	1.95659e-13	2.0744e-18
TIAL1	0.40815	0.5783	2.05785e-23	0
hnRNPC	0.53945	0.49973	1.05658e-42	5.11375e-35
UPF1	−0.18416	−0.15503	1.43503e-05	0.0003
PVT1 protein targets				
PTB	0.21781	0.3321	2.61794e-07	3.44464e-15
eIF4AIII	0.34272	0.41381	1.5088e-16	1.82621e-23
FUS	0.14317	0.23924	0.0007	2.24886e-08
SFRS1	0.32099	0.34682	1.34165e-14	1.64563e-16
U2AF65	0.28095	0.34364	2.12424e-11	3.21989e-16
TIA1	0.29682	0.17857	1.31293e-12	3.38048e-05
TIAL1	0.36842	0.43761	4.64078e-19	2.42026e-26
hnRNPC	0.41684	0.4676	1.90385e-24	2.61554e-30
NEAT1 protein targets				
IGF2BP3	−0.23744	NS	1.84153e-08	NS
TNRC6	0.63612	0.57187	0	0
eIF4AIII	−0.24738	− 0.21657	4.37988e-09	4.45995e-07
DGCR8	0.47992	0.40956	6.47436e-33	5.65318e-23
FUS	0.15949	0.28231	0.0001	3.18582e-11
C22ORF28	−0.41602	−0.39928	2.39532e-24	8.11096e-22
EWSR1	0.45642	0.38323	1.49592e-29	4.32566e-20
FUS-mutant	0.15949	0.28231	0.0001	3.18582e-11
TAF15	0.35774	0.30791	5.4777e-18	3.61613e-13
TIA1	0.39134	0.14772	1.68868e-21	0.0006
hnRNPC	−0.30402	−0.30302	3.50144e-13	8.79981e-13
UPF1	0.26028	0.23562	6.17422e-10	3.69822e-08
TDP43	0.35409	0.27796	1.24663e-17	6.51646e-11
TUG1 protein targets				
HuR	0.23073	0.24185	4.68727e-08	1.56196e-08
PTB	0.23828	0.36496	1.6354e-08	3.08972e-18
IGF2BP1	0.20216	0.20823	1.83405e-06	1.23854e-06
IGF2BP2	0.11891	0.29403	0.005	4.32685e-12
IGF2BP3	0.106	0.24703	0.01304	7.48794e-09
PUM2	0.37119	0.40428	2.41171e-19	2.2431e-22
TNRC6	0.61159	0.49489	0	2.81834e-34
DGCR8	0.65857	0.56409	0	0
FMRP	0.26472	0.1925	3.06394e-10	7.61715e-06
FXR1	0.20584	0.37095	1.17509e-06	7.86123e-19
FUS	0.28187	0.29685	1.8177e-11	2.64246e-12
MOV10	0.28645	0.17153	8.25055e-12	6.88368e-05
ZC3H7B	0.44562	0.40705	4.31803e-28	1.09058e-22
EWSR1	0.59372	0.52829	0	1.20774e-39
FUS-mutant	0.28187	0.29685	1.8177e-11	2.64246e-12
SFRS1	0.42337	0.3621	3.04287e-25	5.87589e-18
U2AF65	0.10951	0.21837	0.01	3.55695e-07
hnRNPC	−0.21224	−0.1016	5.32579e-07	0.0188867
UPF1	0.3765	0.41283	6.73768e-20	2.37266e-23
TDP43	0.5917	0.4423	0	6.1369e-27

## Discussion

Identification and characterization of novel diagnostic and prognostic biomarkers is expected to improve NSCLC patients’ outcomes. The tissue- or cell-specific expression profile of lncRNAs potentiates them as appropriate biomarkers in this regard [3]. In the present study, we evaluated expression pattern of seven lncRNAs in NSCLC samples and their matched ANCTs and showed a gender specific pattern of lncRNA dysregulation in tumoral tissues. *NEAT1* has been significantly over-expressed in NSCLC tissues obtained from male subjects compared with the corresponding ANCTs but not in female subjects. *NEAT1* has been among three lncRNAs with significant over-expression in plasma samples of NSCLC patients [14]. Moreover, *NETA1* over-expression in NSCLC tissues has been demonstrated in a cohort of 125 patients with significant correlation between its expression levels and patient, lymphatic metastasis, vascular invasion and clinical TNM stage [21]. Our data is in line with the results of these two studies in the terms of *NEAT1* over-expression. However, lack of correlation between expression levels of this lncRNA and clinicopathologic data of patients can be at least partly explained by the relative small sample size of the current study.

We also detected significant down-regulation of *FAS-AS1* in NSCLC tissues obtained from both males and females subjects compared with the corresponding ANCTs. This lncRNA has an inhibitory role in alternative splicing of Fas to produce soluble Fas receptor (sFas) in lymphomas. Ectopic expression of *FAS-AS1* leading to down-regulation of sFas has been suggested as a treatment modality in lymphoma [22]. Although the function of this lncRNA has not been assessed in lung cancer cells yet, a previous study has shown the co-expression of Fas and Fas ligand (FasL) in lung cancer cell lines and the apoptotic effect of agonistic anti-Fas antibody in these cells [23]. Future studies are needed to explain the role and status of *FAS-AS1* in regulation of Fas in lung cancer cells.

Moreover, we demonstrated significant down-regulation of *TUG1*, *GAS5*, *THRIL* and *HOTAIRM1* in tumoral tissues obtained from male subjects compared with the corresponding ANCTs. *TUG1* down-regulation has been recently demonstrated in NSCLC tissues obtained from Taiwanese patients [24]. More importantly, they observed a more significant down-regulation of this lncRNA in samples obtained from male patients [24] which is in accordance with our data. *GAS5* has been regarded as a tumor suppressor in NSCLC whose expression was significantly lower in tumoral tissues compared with ANCTs. Such down-regulation has been correlated with TNM stage but not tumor size, lymph node metastasis, age, gender, differentiation and histology type in NSCLC [25]. Consequently, our data regarding gender-specific down-regulation of *GAS5* is not supported by the result of this study. *THRIL* is an lncRNA with regulatory role on TNFα expression and the consequent innate immune response [26]. Although the role of this lncRNA in carcinogenesis has not elaborated yet, the observed down-regulation of it in NSCLC warrants future studies to explain its participation in this kind of human malignancy. Finally, *HOTAIRM1* is a principal regulator of myeloid cell development by targeting HOXA1. *HOTAIRM1* over-expression in myeloid-derived suppressor cells (MDSCs) results in down-regulation of the expression of suppressive molecules in these cells. On the other hand, *HOTAIRM1* levels were shown to be down-regulated in the peripheral blood cells of lung cancer patients compared to those of healthy controls [27]. Consequently, the observed down-regulation of this lncRNA in tumoral tissues of male patients is in line with the previous studies regarding the role of this lncRNA in the pathogenesis of cancer.

Although we assessed expression profile of some lncRNAs in NSCLC using quantitative real time PCR, it is anticipated that computational modeling would be used in near future for the identification of potential NSCLC-related lncRNAs or microRNAs. Computational models would facilitate selection of the most promising candidates for further laboratory investigation so decreasing the labor of the biological researches [28]. The availability of lncRNA-related databases such as those demonstrating annotation of lncRNAs sequences or structures as well as the experimentally validated lncRNA–disease associations or interactions has facilitated this process [29]. Perhaps one of the most important features of these computational models for detection of possible disease-related lncRNAs is possibility of application of a certain model in similar disorders as similar diseases are expected to be linked with functionally comparable lncRNAs [30]. Two recently developed tools for prediction of novel miRNA-disease associations have been shown to be effective and powerful tools for such propose in a wide range of human malignancies [31, 32].

In addition, we demonstrated significant correlations between relative expression levels of lncRNAs especially within tumor tissues and in male subgroup. Such correlations might imply the presence of a single regulatory mechanism for expression of these lncRNAs. Future studies are needed to clarify such mechanism. We also assessed the accuracy of expression levels of these genes in lung cancer diagnosis and demonstrated the best values for *GAS5*. By plotting ROC curves to evaluate the ability of lncRNAs expression to improve the prediction of lung cancer, *GAS5* transcript levels had more than 80% specificity and sensitivity in this regard. On the other hand, *TUG1*, *FAS-AS1*, *HOTAIRM1* and *THRIL* have been demonstrated to be specific markers despite their low sensitivity. Based on these results we recommend future evaluation of this panel of markers in larger samples sizes of NSCLC patients.

Finally, we evaluated target genes of these lncRNAs at both mRNA and protein levels in lung cancer using online tools. We demonstrated involvement of these targets in a number of molecular/signaling networks most of them being recognized as cancer hallmarks. Most importantly, the interactive network between lncRNAs and their targets was shown to participate in different aspects of expression regulation including gene silencing by RNA, regulation of translation, mRNA processing, RNA splicing and posttranscriptional regulation of gene expression.

## Conclusions

In brief, in the present study we demonstrated dysregulation of seven lncRNAs in NSCLC tissues compared with the corresponding ANCTs. Such observations underscore the role of these lncRNAs in the pathogenesis of lung cancer and suggest them as possible biomarkers for this malignancy. Future studies are needed to confirm the results of the current study in larger sample sizes to elaborate their role as cancer biomarkers.

## Abbreviations

ANCTs: Adjacent non-cancerous tissues
AUC: Area under curve
FAS-AS1: Fas-antisense 1
GAS5: Growth Arrest Specific 5
GO: Gene Ontology
HOTAIRM1: HOXA transcript antisense RNA myeloid-specific 1
KEGG: Kyoto Encyclopedia of Genes and Genomes
NEAT1: PVT1, Nuclear Paraspeckle Assembly Transcript 1
NSCLC: Non-small cell lung cancer
ROC: Receiver operating characteristic
THRIL: TNFα and hnRNPL related immunoregulatory LincRNA
TUG1: taurine upregulated gene 1
